# Study of thymus volume and density in COVID-19 patients: Is there a correlation in terms of pulmonary CT severity score?

**DOI:** 10.1186/s43055-022-00917-7

**Published:** 2022-11-08

**Authors:** Banu Alicioglu, Murat Bayav

**Affiliations:** Radiology Department, Faculty of Medicine, Zonguldak Bulent Ecevit University, 67100 Kozlu, Zonguldak, Turkey

**Keywords:** COVID-19, Pneumonia, Thymus gland, Thymus hyperplasia, Tomography, X-ray computed

## Abstract

**Background:**

Thymus has a pivotal role in combating infectious diseases. Although some reviews have been published about its critical role in COVID-19, there is not enough research. In this study, the size and density of thymus related to computed tomography pulmonary severity score (CT-SS) were researched.

**Results:**

A total of 196 patients were analyzed with a mean age of 52.54 ± 18.78 years; 97 (49.5%) of them were RT-PCR (−) and 99 (50.5%) were RT-PCR (+). Within RT-PCR (+) group 62 (62.6%) of them had pneumonia with a mean CT-SS of 9.37 ± 8.83; within RT-PCR (−) group 20 (20.6%) of them had pneumonia with the mean CT-SS of 12.00 ± 10.18. CT-SS had moderate negative correlation with thymus volume and thymus maximum diameter in patients having nodular-type thymus (*R* = −0.591, *P* = 0.02; *R* = −0.515, *P* = 0.049, respectively). Homogenous fat infiltration was more commonly seen in RT-PCR (−) group while reticular and nodular types were commonly seen in RT-PCR (+) group (*p* = 0.015). The mean volume and maximum diameter of thymus were statistically significantly higher in RT-PCR (+) group (*p* = 0.027 and *p* = 0.048, respectively).

**Conclusion:**

This study showed the higher thymic volume and maximum diameter and more involution in COVID-19 patients. CT-SS had a moderate negative correlation with thymus volume and thymus maximum diameter. Pneumonia was more frequent in COVID patients, but mean CT-SS of the non-COVID cases was higher.

## Background

Novel coronavirus disease 2019 (COVID-19) caused by severe acute respiratory syndrome coronavirus 2 (SARS-CoV-2) has brought on pandemic persevering for more than two years. Currently, the pathogenesis and clinical course of COVID-19 is still not fully elucidated; but it is known that the disease starts with viral invasion phase, followed by dysregulated immune response then proceeds to multiple organ damage. The clinical spectrum of the patients with SARS-CoV-2 infection is in a range from no symptoms to critical illness even to death. The overall mortality rate of COVID-19 is estimated at about 3%, but the mortality rate of critical patients is as high as 61.5% [1]. Patients with underlying comorbidities (≥ 65 years-old age, cardiovascular disease, chronic lung disease, diabetes, malignancy, obesity, chronic kidney disease, smoking addiction and immunosuppression) are at a higher risk of progressing to severe COVID-19. Due to rapid progression of the disease, healthcare providers follow risky patients closely until clinical recovery is achieved. Therefore, additional prognostic factors are needed to deal with the patients. Lymphopenia can predict pneumonia development and progression to respiratory failure in the patients. At the early stages of infection, elevated plasma levels of cytokines and C-reactive protein (CRP) may be the markers of forthcoming severe illness, suggesting that hypercytokinemia-related immunopathology has an essential role in severe COVID-19. Since the beginning of the pandemic, computed tomography (CT) has played a great role in fighting the disease. Many studies agree that the severity of COVID-19 in the initial chest CT images was a strong predictor of critical illness and CT-severity score (CT-SS) was widely applied as a criterion for triage of patients who require hospitalization [2].

The thymus is an integral of lymphoid system. It produces circulating T lymphocytes responsible for cellular immunity. The thymus has an age-related morphology. Its size reaches maximum until adolescence. After that, it withstands involution despite its ongoing T lymphocyte generation. However, immune senescence or thymic aging causes inflammatory conditions that trigger chronic diseases of comorbidities in elders (atherosclerosis, hypertension, chronic obstructive lung diseases, arthritis). Similarly, these comorbidities consist increased risk of severe COVID-19 and mortality [3]. Chest CT scans have been routinely performed in the evaluation of patients to detect pulmonary involvement; on the other hand, thymus having an elementary role to regulate immune system has not been widely investigated in radiologic studies.

We aimed to study radiologic features of thymus in RT-PCR (reverse transcription polymerase chain reaction) confirmed COVID-19 patients’ initial CT scans and find a relationship in terms of pulmonary involvement.

## Methods

Local ethical committee approved the study (approval date and number: 15.04.2020; 2020/08). Due to the retrospective nature of the study, pandemic restrictions informed consent was waived by the ethical committee. All the non-contrast-enhanced chest CT scans done in our Radiology Department were retrospectively scanned from the hospital information system between 16 March 2020 and 30 May 2020 (the first peak of the pandemic). There was a total of 504 patients, among them, the patients clinically diagnosed with upper or lower respiratory system infections were determined from their files. A total of 106 patients confirmed by using a real-time SARS-CoV-2 RT-PCR throat swab were selected. The age and gender-matched control group was selected (101 patients) between patients having negative RT-PCR test results. Initial chest CT scans of the patients were included. Exclusion criteria were previous surgery, radiotherapy or malignancy related to anterior mediastinum. 7 of RT-PCR positive and 4 of RT-PCR negative patients were excluded due to recent bypass surgery. Three patients in RT-PCR negative group were excluded because of mesothelioma invading mediastinum. Finally, 97 RT-PCR negative and 99 RT-PCR positive patients were included in the study.

### Imaging technique

All patients underwent scanning with the following two scanners: Toshiba Aquilon 16 detectors, Somatom Spirit (Siemens Healthcare), 2 detectors. The acquisition parameters were set at 120 kVp; 100–200 mAs; pitch, 0.75–1.5; and collimation, 0.625–5 mm. All imaging data were reconstructed by use of a medium sharp reconstruction algorithm with a slice thickness of 5–10 mm. CT images were acquired at full inspiration with the patient in the supine position. The images were viewed with lung parenchymal (width: 1500 HU; level, −700 HU) and mediastinal (width, 350 HU; level, 40 HU) settings.

Chest CT images of the patients were analyzed by a board-certified radiologist with 22 years of experience for severity index. Semiquantitative CT-SS system was applied in calculations based on the degree of involvement of each lung segment as 0%, (0 points), < 25% (1 point), 26–50% (2 points), 51–75% (3 points), and 76–100% (4 points). The CT-SS was quantified by summing the 10 segments for both lung indices (maximum 40 points) as defined in the previous literature (2).

**Fig. 1:**
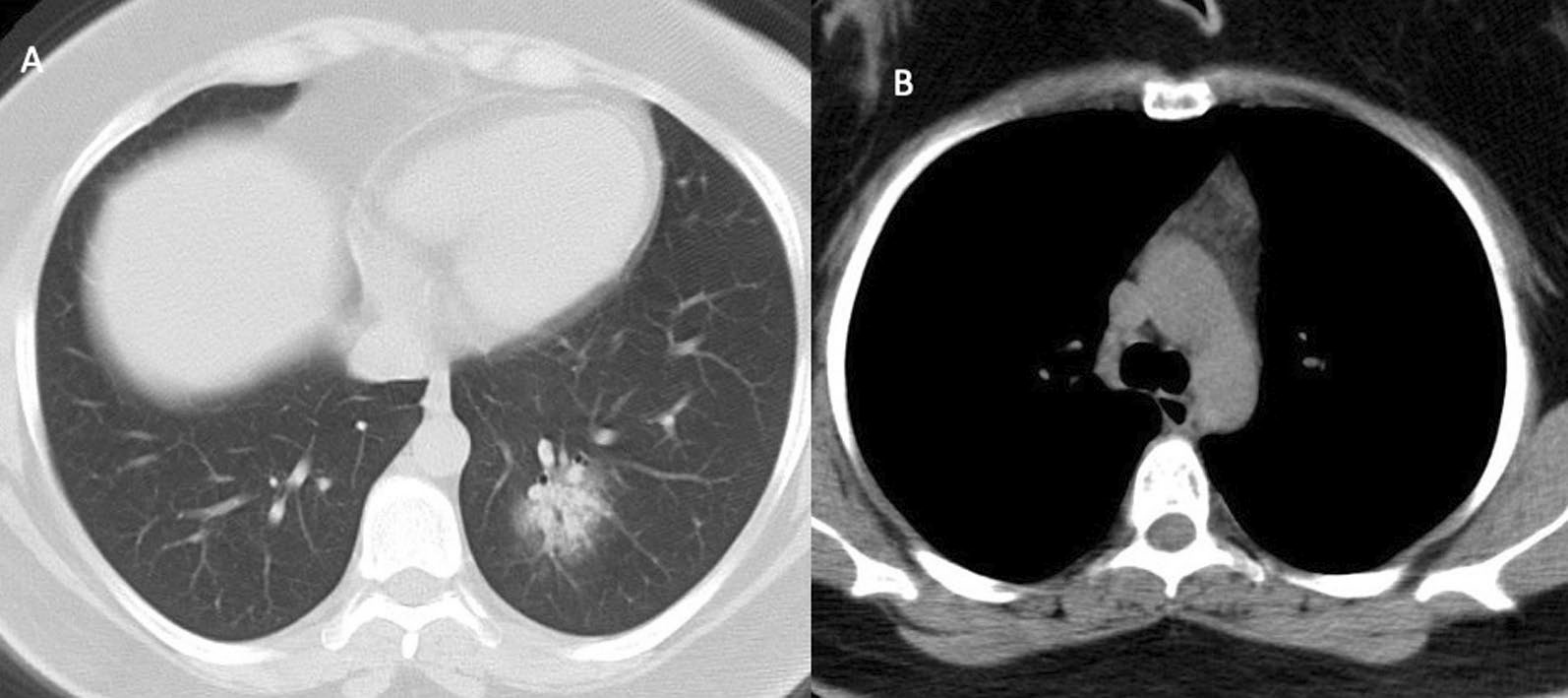
26-year-old female COVID-19 (+) patient with CT-SS 1 (**A**) and nodular-type thymus (**B**)

**Fig. 2:**
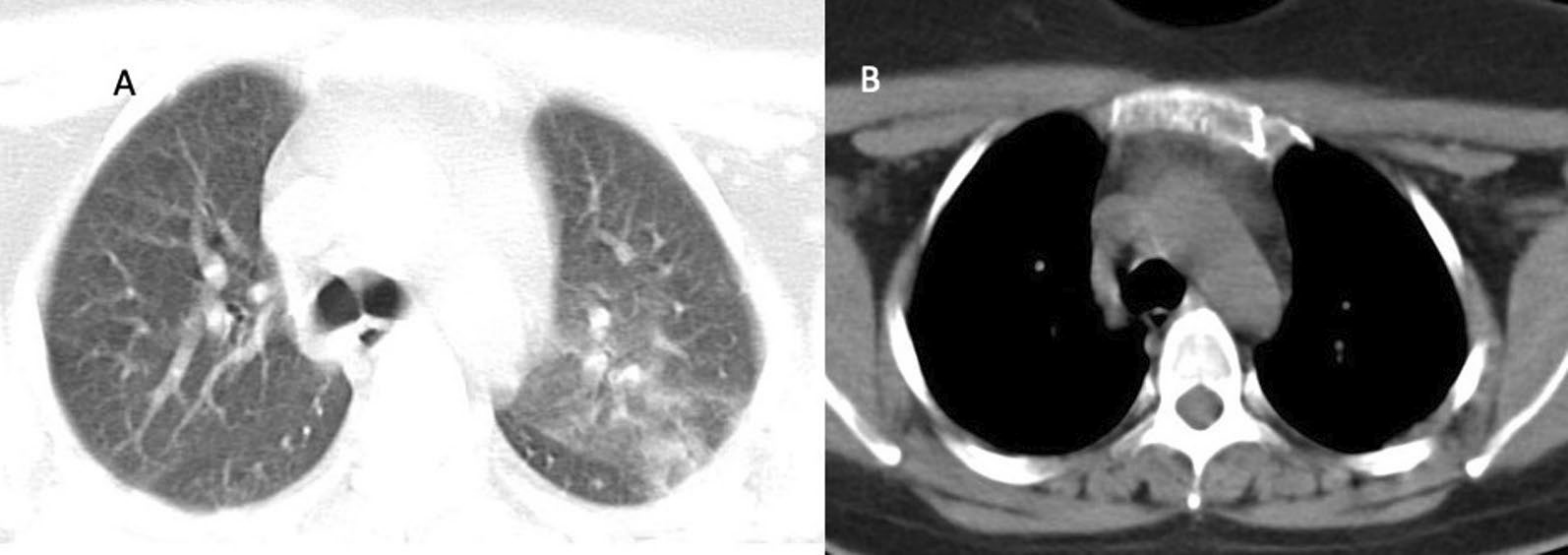
29-year-old female COVID-19(+) patient with CT-SS 12 (**A**) and nodular-type thymus (**B**)

**Fig. 3:**
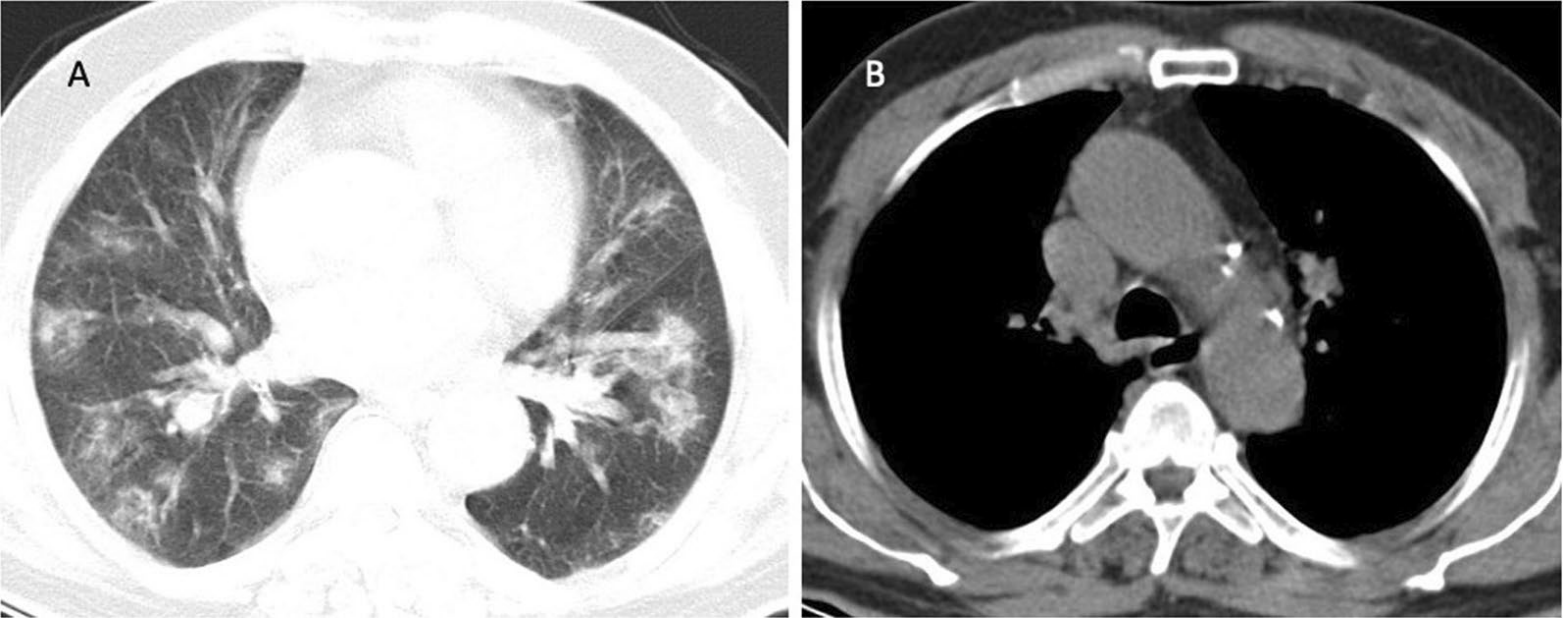
66-year-old male COVID-19 (+) patient with CT-SS 20 (**A**) and fatty thymus (**B**)

**Fig. 4:**
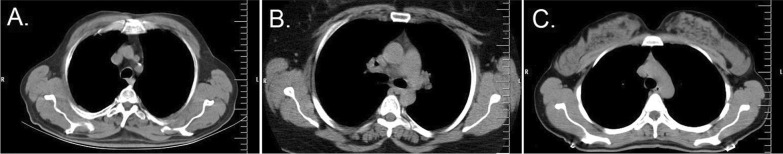
Homogeneous fat; which was entirely fat infiltration to anterior mediastinum (**A**), reticular type; fatty infiltration of thymus containing < 50% thymus tissue without well-defined contours (**B**) and nodular type; which had < 50% fat infiltration with well-defined contours (**C**)

**Fig. 5:**
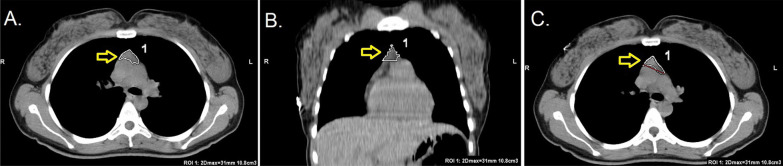
Measurements of thymus with auto contour tool; **A** and **B** shows automated and manually fine tuned thymus contours. **C** Automatically measured maximum diameter

The thymus-type analysis from the CT images was done by a radiologist with 3 years of experience. Images were analyzed and evaluated on HP z4 Workstation and GE AW Volume Share 7™ software (Chicago, Illinois, USA). Thymus types were categorized as follows: (i) homogenous fat, which was entirely fat infiltration to anterior mediastinum, (ii) reticular type, fatty infiltration of thymus containing less than 50% thymus tissue without well-defined contours and iii) nodular type, which had less than 50% fat infiltration with well-defined contours (Figs. 1, 2, 3). Patients having nodular-type thymus were analyzed also for thymus volume, maximum diameter and mean density with the aid of 3D volume viewer application and auto-contour tool (Figs. 4, 5).

### Statistical analysis

Continuous data were shown as mean ± SD, categorical data were shown as numbers (percentage). Shapiro–Wilks test was used for exploring the normality of data. Independent samples t-test was used for comparison of means between groups. Chi-square test and Spearman’s correlation coefficient were used to compare cross-tables and correlation of continuous variables, respectively. Statistical analyses were done with SPSS version 25 (IBM Corp, Armonk, NY, USA) software. < 0.05 *p* values were considered statistically significant.

## Results

A total of 196 patients were analyzed with a mean age of 52.54 ± 18.78 years. Of these 196 patients, 97 (49.5%) of them were RT-PCR negative and 99 (50.5%) of them were RT-PCR positive for COVID-19. Demographic and CT features of the patients groups are summarized in Table 1.

**Table 1:** Summary of the patients demographic data and CT measurements

		All the patients (RT-PCR− and +)	RT-PCR+	RT-PCR−	*p*
Number of the patients, (%)		196	99 (50.5%)	97 (49.5%)	> 0.05
Age		52.54 ± 18.78	53.14 ± 19.03	51.93 ± 18.59	0.652
Pneumonia	Absent	114 (58.2%)	77 (39.3%)	37 (18.9%)	< 0.001^#^
	Present	82 (41.8%)	20 (10.2%)	62 (31.6%)	
CT-SS (mean, SD, min–max)		10.01 ± 9.18 (1–37)	9.37 ± 8.83 (1–37)	12.00 ± 10.18 (2–34)	0.268
The mean volume of thymus (mean, SD) (mL)		10.16 ± 8.63	12.34 ± 10.09	6.65 ± 3.59	0.027*
The mean maximum diameter of the nodular-type thymus (mean, SD), mm		31.11 ± 11.89	33.79 ± 13.26	26.78 ± 7.78	0.048*
The mean thymus density (mean, SD) HU		4.12 ± 32.59	2.16 ± 33.21	7.27 ± 32.26	0.605

*SD* standard deviation, *HU* Hounsfield unit

#Data were shown as n (%), *χ*^2^ = 35.53, *p* < 0.001

*Statistically significant *p* < 0.05

The mean age of RT-PCR positive group was 53.14 ± 19.03 years old and RT-PCR negative group was 51.93 ± 18.59 years old with no significant difference between the two groups (*p* = 0.652). There were 46 (23.5%) RT-PCR positive, 48 (24.5%) RT-PCR negative men and 53(27%) RT-PCR positive, 49 (25%) RT-PCR negative women with no statistically significant difference between groups (*p* = 0.672).

Within RT-PCR positive group, 62 (62.6%) of them had pneumonia with the mean severity score of 9.37 ± 8.83 ranging from 1 to 37 and 37 (37.4%) of them had no pneumonia. Within RT-PCR negative group, 20 (20.6%) of them had pneumonia with the mean severity score of 12.00 ± 10.18 ranging from 2 to 34 and 77 (79.4%) of them had no pneumonia. Pneumonia was more commonly seen in RT- PCR positive group.

Of all 196 patients, the mean semi-quantitative CT-SS was 10.01 ± 9.18 (1–37) in the total of 82 (41.8%) patients having pneumonia. There was no pneumonic infiltration in 114 (58.2%) patients, so they had zero semi-quantitative CT-SS. CT-SS had moderate negative correlation with thymus volume and thymus maximum diameter in patients having nodular-type thymus (*R* = −0.591, *p* = 0.02; *R* = −0.515, *p* = 0.049, respectively). There was no statistically significant correlation between the thymus density and the CT-SS (*R* = 0.318, *p* = 0.248).

Of all 196 patients, thymus types were homogenous fat infiltration in 85 (43.4%) patients, reticular in 64 (32.7%) patients and nodular in 47 (24%) patients. Distribution of thymus types in RT-PCR positive and negative patient group is shown in Table 2. Homogenous fat infiltration was more commonly seen in RT-PCR negative group while reticular and nodular types were commonly seen in RT-PCR positive group (*χ*^2^ = 8.36, *p* = 0.015).

**Table 2:** Distribution of thymus type according to RT-PCR test result

		RT-PCR	
		Negative	Positive
Thymus type	Homogenous fat	52 (26.5%)	33 (16.8%)
	Reticular	27 (13.8%)	37 (18.9%)
	Nodular	18 (9.2%)	29 (14.8%)

Data were shown as *n* (%), *χ*^2^ = 8.36, *p* = 0.015

The volume, maximum diameter and mean density of the nodular-type thymus had weak negative correlation with the patient age (*R* = −0.298, *P* = 0.042; *R* = −0.313, *P* = 0.032; *R* = −0.288, *P* = 0.49, respectively).

The mean volume of the nodular-type thymus was 10.16 ± 8.63 (0.79–41.9) mL for all patients. The mean volume of thymus was 12.34 ± 10.09 mL in the RT-PCR positive group and 6.65 ± 3.59 mL in the RT-PCR negative group, which was statistically significantly higher in RT-PCR positive group (*p* = 0.027). The mean maximum diameter of the nodular-type thymus was 31.11 ± 11.89 (11–57) mm in all patients. Maximum diameter of thymus was 33.79 ± 13.26 mm in the RT-PCR positive group and 26.78 ± 7.78 mm in the RT-PCR negative group, which was statistically significantly higher in RT-PCR positive group (*p* = 0.048). The mean thymus density was 4.12 ± 32.59 HU. No statistically significant difference was seen in mean thymus density in RT-PCR test groups (*p* = 0.605).

Among all patients (either RT-PCR positive or negative), the mean volume of thymus was 10.37 ± 10.52 mL in the patients having pneumonia and 10.06 ± 7.78 mL in the patients having zero CT-SS (no pneumonic infiltration). Maximum diameter of thymus was 30.2 ± 12.83 mm in the patients having pneumonia and 31.53 ± 11.61 mm in the patients with no pneumonic infiltration. The mean thymus density was −2.91 ± 29.73 HU in the patients having pneumonia and 7.42 ± 33.79 HU in the patients with no pneumonic infiltration. There was no statistically significant difference in all the measurements in groups (*P* = 0.911, *P* = 0.725 and *P* = 0.316, respectively) (Table 3).

**Table 3:** Summary of the thymus features between the patients with pneumonia and without pneumonia in all of the study group (RT-PCR positive and negative)

	All the patients (RT-PCR positive and negative)		
Thymus	Pneumonia+	Pneumonia**−**	*p*
Volume (mL)	10.37 ± 10.52	10.06 ± 7.78	*0.911*
Max diameter (mm)	30.2 ± 12.83	31.53 ± 11.61	*0.725*
Density (HU)	−2.91 ± 29.73	7.42 ± 33.79	*0.316*

Statistically significant *p* < 0.05

## Discussion

Our cohort reflects early CT features of the thymus in the first peak of the pandemic. The volume and maximum diameter of the thymus were found to be higher in RT-PCR positive patients compared to non-COVID patients. Especially thymic volume was twofold higher. The result was congruent with Cuvelier et al.’s; they first observed that 66% of the COVID patients in intensive care unit had thymus enlargement in their initial chest CT scan [4]. Even after the administration of mRNA vaccination (BNT162B2, mRNA-1273) against SARS-CoV2, can result in thymic hyperplasia with increased uptake of FDG in 18F-FDG PET/CT scan [5]*.* Thymic enlargement is thought to be a response to profound lymphopenia that is frequently seen in severe COVID patients. This suggests that the T cell response may be necessary for SARS-CoV-2 clearance [1]. Nevertheless, increased thymic volume cannot be attributed only to increased generation and exporting of mature T cells into the blood; because the most accurate marker of thymic activity is measurement of T cell receptor excision circles (products of chromosomal rearrangements that occur at T cell receptor loci during T cell development in the thymus). Thymus may enlarge following recovery from recent stresses (autoimmune diseases, burn, radiotherapy, chemotherapy, corticosteroid treatment, lymphopenic patients infected by human immunodeficiency virus (HIV) with maintained naïve T cell counts or after antiretroviral therapy). Consequently, the thymus may become atrophic; but it may also recover to its original size after ceasing of the stress [4, 6].

In spite of the increase in the use of high-resolution imaging systems, evolution of the thymus related to aging or acute-chronic diseases is neglected. CT density and volume of the thymus was first studied in the HIV-1-infected patients [7, 8]. Thymic density was found to be highly correlated with circulating naive T cell subpopulations in both HIV-1–seropositive and HIV-1–seronegative adults and abundant thymic tissue (similar to nodular thymus type in our study) was higher in younger HIV seropositive adults suggesting that the thymus don’t show similar functionality in all adult HIV-1 patients [7].

Pulmonary involvement degree detected by CT is reported to be a valuable tool to predict the severity of disease in clinically moderate patients on first admission in COVID-19 patients [9–13]. We found that CT-SS had a moderate negative correlation with thymus size. In severe COVID patients, massive infiltration of macrophages in the lungs and increased T cell response to the virus are responsible in immune response [14]. Acute COVID-19 infection may also lead thymic involution because of acute loss of cortical thymocytes and reduced output of naïve T cells to the periphery. As a contradiction to our study, Cuvelier et al. [4] observed thymic enlargement was associated with more severe pulmonary involvement in intensive care patients. The mortality rate was lower in patients with thymic enlargement and the overall survival was negatively correlated with T cell precursors proliferation in the thymus. So they concluded that thymic hyperplasia in COVID patients was a favorable adaptation against virus-induced lymphopenia. The difference between the two studies should be explained by the heterogeinity of the patients selection, Cuvelier et al. studied severe COVID patients while our study was consisted of mild-to-severe COVID cases.

Old age and comorbidities are risky for severe COVID-19. Thymic involution during aging leads to immune senescence and triggers an inflammatory condition that is also common in pathogenesis of diseases related to aging (hypertension, cardiovascular disorders, chronic obstructive lung disease, arthritis, malignancy). In severe COVID-19 patients, lymphopenia with reduction in CD4+ and CD8+T cells, cause activation and dysfunction of lymphocyte, increase in circulating neutrophils, dysfunction and loss of monocytes, reduced plethora and dysfunction in oligodendritic cells therefore natural killer cells are seen. Then systemic inflammatory cytokine levels (interleukin IL-6 and IL-1) are increased. In the end, over inflammation and immunosuppression develop due to dysregulated immune response. For this reason, the cellular immune response during mild disease can be considered as a probable progression to severe COVID-19 [15, 16].

Pneumonia was threefold higher in COVID patients (62.6% vs. 20.6%). However, mean CT-SS of the non-COVID cases was higher (9.37 ± 8.83 vs. 12.00 ± 10.18) ranging from 2 to 34 and 77 (79.4%) of them had no pneumonia. The reason of higher incidence of pneumonia (that is also named acute lung injury) in COVID-19 patients is usual association of coagulopathy resulted by epithelial damage vascular thrombosis, massive pulmonary edema and infarctions [17]. Unfortunately because of missing laboratory analysis of microbiological agent and immune status of non-COVID cases, we cannot discuss the result.

RT-PCR (+) patients have had significantly more involution of the thymus (26.5%); while reticular thymus was detected in 13.8, and 9.2% of them had nodular thymus. During thymic involution process, the epithelial content atrophies, causing adipose tissue to increase within scattered small lymphocytes that manifested as decrease in the size and weight of the gland [3]. Thymic density was less in COVID pneumonia cases but no statistically significant correlation was found between the thymus density and the CT-SS. This can be related to the fact that both nodular thymus group and pneumonia patient groups were heterogeneous; as they had different age and gender or RT PCR test results may have spoiled the correlation. Also, obesity may be an important factor for thymic involvement in younger people [18]. Since the beginning of the pandemic, it was obvious that obesity was a risk factor for severe COVID-19. Obesity constricts the mechanisms regulating T-cell generation by accelerating the age-related thymic involution leads type 2 diabetes, cardiovascular disease and cancer [19, 20]. However, the reason of increased frequency of reticular/nodular thymus in COVID patients couldn’t be explained due to lack of clinical or laboratory data in our cohort.

The main strengths of the present study are that the study cases consist of the ones from the first peak of the pandemic when there was no mutation of the SARS-CoV-2 or no vaccination to modulate humoral immune response or no specific drug had been developed yet. Because RT-PCR tests were not available or the results were too slow, at that time the patients had to be scanned with CT to enable rapid filiation. Therefore, it was possible to find high number of patients as well as controls due to the coincidence with seasonal influenza.

The major limitation of the study is the lack of laboratory parameters (especially T-lymphocyte and leukocyte, monocyte, fibrinogen, D-dimer, C-reactive protein, erythrocyte sedimentation rate), and clinical features, comorbities and prognosis of the patients. The lack of microbiological agent identification of the non-COVID patients is another limitation. We don’t have body mass index data to find out a relationship between obesity, thymic involution and pulmonary involvement. CT measurements were done by single observer, intra/interobserver variances are not possible. 5–10 mm of slice thickness made step artifacts during the measurement of thymus volume. Volume measurement of the thymus was only possible in nodular and reticular-type thymuses because it is not possible to detect thymic borders in fatty involvement. Initial chest CT of the patients were studied, but what stage were the patients or duration of the symptoms were unknown. It would be better to know thymus volume and density before the start of infection or after recovery to conclude objective alterations of the thymus due to acute infection. Therefore, we cannot assume the thymic CT features are cause or effect of the disease process.

## Conclusions

Despite its critical role in immune system, the thymus tissue is often neglected on thorax CT evaluations. The thymic volume and maximum diameter as well as fatty infiltration are higher in COVID-19 patients. Pneumonia was threefold higher in COVID patients, but mean CT-SS of the non-COVID cases was higher. CT-SS had a moderate negative correlation with thymus size. Further clinic and laboratory investigations in thymus and infections are needed to clearly understand the relationship between the two. Thymic evolution should be considered as a potential biomarker in evaluation of chronic and acute disorders. Further studies are needed to reseach evolution of thymus related to acute or chronic diseases together with immunological responses.

## Data Availability

The datasets used and/or analyzed during the current study are available from the corresponding author on reasonable request.

## Abbreviations

COVID-19: Coronavirus disease 2019
SARS-CoV-2: Severe acute respiratory syndrome coronavirus-2
CT: Computed tomography
CT-SS: Computed tomography severity score
RT-PCR: Reverse transcription polymerase chain reaction
HIV: Human immunodeficiency virus
